# Building Collaborative Health Promotion Partnerships: The Jackson Heart Study

**DOI:** 10.3390/ijerph13010025

**Published:** 2015-12-22

**Authors:** Clifton C. Addison, Brenda W. Campbell Jenkins, Darcel Odom, Marty Fortenberry, Gregory Wilson, Lavon Young, Donna Antoine-LaVigne

**Affiliations:** Jackson Heart Study, School of Public Health, Jackson State University, 350 W. Woodrow Wilson Drive, Jackson, MS 39213, USA; brenda.w.campbell@jsums.edu (B.W.C.J.); darcel.thigpen@jsums.edu (D.O.); marty.fortenberry@jsums.edu (M.F.); gregory.wilson@jsums.edu (G.W.); lavon.young@jsums.edu (L.Y.); donna.antoine-lavigne@jsums.edu (D.A.-L.)

**Keywords:** community-based partnership and collaboration, community-based research (CBPR)

## Abstract

Building Collaborative Health Promotion Partnerships: The Jackson Heart Study. *Background:* Building a collaborative health promotion partnership that effectively employs principles of community-based participatory research (CBPR) involves many dimensions. To ensure that changes would be long-lasting, it is imperative that partnerships be configured to include groups of diverse community representatives who can develop a vision for long-term change. This project sought to enumerate processes used by the Jackson Heart Study (JHS) Community Outreach Center (CORC) to create strong, viable partnerships that produce lasting change. *Methods:* JHS CORC joined with community representatives to initiate programs that evolved into comprehensive strategies for addressing health disparities and the high prevalence of cardiovascular disease (CVD). This collaboration was made possible by first promoting an understanding of the need for combined effort, the desire to interact with other community partners, and the vision to establish an effective governance structure. *Results:* The partnership between JHS CORC and the community has empowered and inspired community members to provide leadership to other health promotion projects. *Conclusion:* Academic institutions must reach out to local community groups and together address local health issues that affect the community. When a community understands the need for change to respond to negative health conditions, formalizing this type of collaboration is a step in the right direction.

## 1. Introduction

The Jackson Heart Study (JHS) is a large, community-based study of 5301 African Americans residing in the tri-county area of Hinds, Madison and Rankin counties in Mississippi. It is very well documented that African Americans in Mississippi communities and the Jackson Heart Study are faced with ongoing health challenges and that there is a large disparity in cardiovascular mortality between Mississippi African Americans and Mississippi Whites or African Americans from other parts of the US [1,2]. To eliminate some of the disparities observed in the African American communities, committed academic-community collaborative and sustained research may be beneficial [3]. Since little has changed in the health status of African Americans through the years, innovative methods and culturally appropriate interventions are needed [4] to reverse the deprivation and disadvantage that are often characteristic of the social landscape for African Americans [5,6]. Many of these factors occurring at the individual and community levels have implications for development of chronic disease and long-term disability [7,8,9].

The JHS Community Outreach Center (CORC) ascribes to a community-based participatory research (CBPR) approach that promotes the equitable involvement of partners in the entire research process, having a voice in the formulation of the project purpose and questions, outputs and outcomes, while contributing expertise, and sharing decision making and ownership [10,11]. For members of the JHS community, this arrangement promises to build community capacity as it enables them to become actively involved in the full range of JHS research-related activities. The principles of CBPR that are particularly important and relevant for the Jackson Heart Study are the following: (1) support for an approach that clearly illustrates cultural relevance in its activities where the JHS communities have the opportunity to define their health priorities and research agenda themselves; (2) willingness to operate in an environment that exudes mutual trust and respect where the JHS and its collaborating partners establish, develop, and openly cultivate respect and trust); (3) allocation of adequate and sustained resources needed for long-term health interventions that are readily made available; and (4) support for sustainable partnerships where there is an agreement for a long-term committed relationship between academic partners and the community partners [10,11,12,13]. The main JHS academic partner with the community is Jackson State University that was awarded a contract with the National Heart, Lung, and Blood Institute (NHLBI) of the National Institutes of Health (NIH) in 2013 to develop the Community Outreach Center (CORC). The JHS CORC is the first center developed within a national study by NHLBI that is dedicated exclusively to community outreach and engagement and operates under the auspices of the Jackson State University (JSU) School of Public Health. For the JHS CORC, community engagement and outreach includes providing avenues to reduce health disparities and engaging the community in health disparities discussions. Effective strategies can only be accomplished by convening ongoing academic-community discussion where community members can exchange ideas and be involved in the decisions that are made regarding strategies needed to address community concerns from the formative stages.

The JHS CORC has infused innovative strategies to engage the public and the scientific community in understanding the risks for development of CVD and accelerating interest in collaborative approaches to reducing the impact of CVD.

To be successful in adequately addressing the objective of community outreach and engagement, the JHS Community Outreach Center (CORC) at Jackson State University sought to, according to the principles of CBPR, create an environment of trust and long-term support of the JHS. This trust building mission was solidified by providing opportunities for substantive community involvement in all phases of the study development, conducting health promotion/education activities, and educating the community and study participants on the progress and processes of the JHS. The CORC staff emphasized that the success of this history-making medical research study depends on sustained involvement by the residents in the tri-county area of Hinds, Madison, and Rankin counties. This type of commitment is necessary to ensure that the academic-community union can effectively lay a strong foundation for establishing improved cardiovascular health within the African-American community [14].

The information presented in this manuscript highlights the key procedures implemented by the JHS CORC to engage the community and the collaborative efforts that inspired the development of the academic-community partnerships. This paper highlights the operations and the actions that were implemented to sustain the Community Health Advisory Networks (CHAN) in all three JHS counties, (Hinds, Madison, and Rankin counties) under the premise that a successful academic-community partnership can increase participation in health education and health promotion activities, empowering community members to take an active role in improving their individual and community health status.

## 2. Methods

One key initial organizational strategy was a two-day Strategic Planning Retreat that was convened to create a vision of expected long-term change. The Strategic Planning Retreat was planned jointly by CHAN leaders and JHS CORC, and attended by selected representatives from the CHANs located in the three JHS counties -Hinds, Madison, and Rankin counties. The Strategic Planning Retreat was also attended by the members of the CORC staff. In deciding the process of engagement, several issues had to be resolved by the partners. They were: 1.identify the problem or the community needs, aligning all activities and decisions with CBPR principles; 2. decide on the expected outcome or the vision for the future; and 3. decide on a strategy for achieving the goals.

The final plan included creating the academic-community mission statement, a tagline, and the establishment of five *CORC Working Groups* charged with developing and implementing action plans, strategies, and steps to allocate resources to address the five specific aims of the CORC that were developed to fulfill the objectives of the JHS. The Working Groups were developed based on needs, suggestions, and input from the community partners, with the expectation that community members would continue to participate and provide input in the operations of the Working Groups that manage the operations of the CORC. The partners decided that the Mission Statement for the JHS CORC would be to foster a welcoming, respectful, and collaborative community-academic environment that promotes health equity through prevention, education, training, and research. This is a critical component of the CBPR principles. The group also decided on a tagline which is “Enriching CommUNITY one heart at a time”.

### 2.1. CORC Logic Models for Effective Academic-Community Partnerships

In order to effectively plan the program of activities and initiate the academic-community partners’ initiatives, it was decided that the CBPR principles and methodologies would be infused into CORC’s overall mission, activities, and evaluation. To ensure successful delivery of its objectives, CORC program administrators sought community input and developed an action plan and a roadmap with clear outcomes and explicit steps to implement an effective academic-community partnership. The result of these deliberations was the creation of a logic model. The plan of action was cultivated using the power of consensus and relying upon the group’s examination of the community’s values and beliefs. This process of collaboration facilitated the academic-community partners’ development of realistic expectations for the resources needed for operation, the visualization of the activities to be initiated, and the vision of changes or results expected to be achieved. A comprehensive logic model was developed to explain the relationships of these elements to the expected outcomes (see Table 1). The intention of this logic model was to guide the overall scientific integrity of the academic-community partnership ensuring that knowledge acquired could be transferred into action that could influence individual and societal lifestyle, behavioral, and structural change [15].

**Figure 1:**
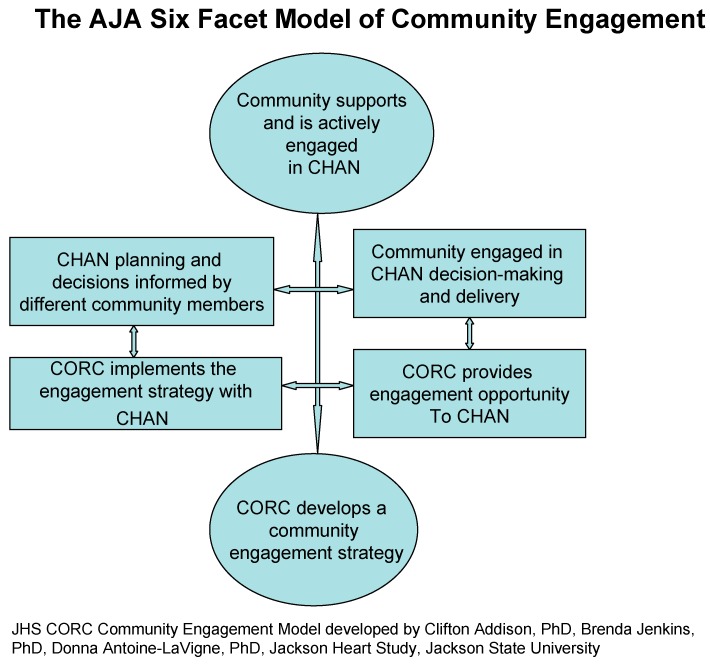
Model to initiate community engagement.

Prior to the development of the Logic Model, the academic-community continuing partnership was solidified with the development of a conceptual model for continuing engagement and outreach with the community partners. The collaboration and interaction between the CORC staff and the CHANs led to development of the Addison-Jenkins-Antoine-LaVigne (AJA) Six-Facet Model of Community Engagement. The AJA model's Six Types of Involvement (see Figure 1) provides specific ideas on how the JHS CORC and community members can best impact community outreach, inspiring community members to participate in decision-making and leadership regarding health promotion and elimination of health disparities. The AJA model was developed by infusing experiences and ideas generated over a period of 13 years of community mobilizing/partnership-building that was the focus of the functions of the JHS Community Partnership Office (CPO). During the first contract period of the JHS (2000–2013), the CPO was infused into the operations of the JHS Coordinating Center and became recognized as a major force in the JHS cohort recruitment and retention efforts [16,17].

**Table 1:** Community outreach center (CORC) logic model 1. Logic model: building institution and community partnership/commitment.

SITUATION: Developing institutional and community commitment to CBPR through facilitating respect for the knowledge and expertise the community brings to the table. Outreach programs and activities are conducted and supported through the Jackson Heart Study (JHS) Community Outreach Center (CORC). The premise is that the community will support what it helps to create.							
Priorities: Building and sustaining relationships with community partners							
INPUTS: JHS CORC, NHLBI, Community Groups							
Outputs		Outcomes-Impact					
Activities	Participants	Short-Term		Medium-Term		Long Term	
What we do	Who we reach	Learning changes	Indicators	Action changes	Indicators	Condition changes	Indicators
Develop a model for assembling an effective community partnership Sponsor seminars, forums, and conferences Address barriers of distrust and cultural insensitivity Disseminate health-related research findings to community-based primary care providers, community members Enhance community health education and awareness using cardiovascular disease-related research training in health education Design media messages and build capacity for community partners	African American scientists, health care providers and scholars Faith and community-based organizations, and other grassroots organizations	Increase awareness, knowledge, and understanding about the JHS and the importance of African American participation Accessibility to this information will further enhance the knowledge of the area providers	List of contacted potential partners List of types of contacts made Outline agreements reached Completed program evaluation List of media activities and training programs List of capacity building activities	Attend JHS- sponsored activities Clinic appointments are scheduled Formation of a coalition, an organized body of diverse individuals, organizations and social and civic groups from Hinds, Madison and Rankin counties JHS committee assignments Implementation of Community Health Advisors Networks (CHAN) leadership trainings, conferences/forums, focus groups, town hall meetings, health fairs	Attendance /Sign-in register Documented list of coalition members Compiled list of committees and assignments List of established CHAN List of trainings, conferences, meetings, health fairs	Sense of partnership and continued participation in JHS activities Continuing participation in Community Partnership activities Elimination of barriers of distrust and cultural insensitivity Substantive community involvement in all phases of the study’s development and implementation Receipt of the JHS newsletter	Transcripts from meetings List of meetings/ appointments scheduled Completion of evaluation instruments demonstrating change in attitudes, beliefs, practices, status Indication of long-term improvements in health status Indication of reduction of CVD risk behaviors, Collecting and utilizing health care and public health material

### 2.2. CORC Theoretical Model for Community Engagement (AJA Model)—JHS CORC Community Engagement Model (Developed by Clifton Addison, PhD; Brenda Jenkins, PhD; and Donna Antoine-LaVigne, PhD (Jackson Heart Study, School of Public Health, Jackson State University)

This model provided a pathway for effective academic-community partnerships and laid the foundation for effective program evaluation. According to the AJA Model, CORC is responsible for providing support to the JHS communities to develop the programs and activities that lead to the organization and management of the Community Health Advisory Networks. The AJA model outlines activities and roles for both the CORC staff and for the JHS CHAN. This is a system that encourages and supports full participatory community involvement efforts. The AJA model proposes that the partners create an official body within their respective communities with specific assignments to manage the day-to-day responsibilities. They operate with a definite plan of action, with clear objectives, roles, schedule of events, timelines, and deadlines.

### 2.3. CORC Evaluation

After infusing the Community-Based Participatory Research (CBPR) principles into the academic-community partnership’s mission, activities, and evaluation, it became necessary to generate practice-based evidence through analysis of the ongoing activities and practices of the partnership. Based on the sequence of activities generated through the logic model, an evaluation plan was developed to document the effectiveness of the activities. The Contact, Initiation, Acceptance, Success, and Continuation Evaluation Framework (CIASC) was developed to guide the evaluation of the CORC CHAN activities. CIASC represents the sequence of activities utilized by the academic-community partners to address the mission and objectives of the partners. The following are the components of the CIASC Evaluation Model, highlighting the areas of CORC CHAN activities that provide a framework for analyzing the partners’ ability and capacity to meet objectives.

1. CONTACT—Develop a framework/model and identify, contact, and interact with potential partners.
2. INITIATE (OUTREACH)—Introduction of CORC/Community planned activities and ensuring performance.
3. ACCEPTANCE (ADOPTION)—Recording partners’ acceptance of the model and agreement to participate and adopt plan of activities.
4. SUCCESS—Documenting results, value, and usefulness of activities.
5. CONTINUATION—commitment to sustainability of activities, long-term preservation of ideals developed.

## 3. Results

Given the need to increase CBPR in the JHS community, the use of CORC CHAN activities was an important community intervention vehicle. Utilizing CBPR principles was crucial for facilitating and encouraging community involvement in health promotion and created the platform for the community partners to work cooperatively to effect a reduction in health disparities and premature development of morbidity and mortality. The JHS CORC has promoted and supported the JHS community's involvement by modeling CBPR, co-participating in health promotion/health education activities, encouraging or prompting health promotion, and providing support. Through the CHAN mechanism, CORC staff, in collaboration with the community partners, has promoted a model of effective academic-community-family-partnerships, through which CORC can maintain contact, continue to build relationships, communicate awareness, and provide support, while serving as a valuable resource for JHS community members. CORC has endorsed, identified, and introduced opportunities for the CHAs to become more involved in the conduct of research. The interaction and collaboration between the JHS CORC and the CHAN have paved the way for a new community interest in research activities.

### The JHS Newsletter as a Vehicle for Community Education/Engagement Events

Partnerships begin with communication, and effective communication must be maintained to facilitate ongoing relationship-building. One key medium of ongoing academic-community communication is the distribution of the Jackson Heart Study *Heartbeat* Newsletter bringing together the community partners for a common purpose. The content of the JHS newsletters usually include the results of research studies examining risk factors relating to the long-term consequences of dietary practices, the impact on health, and policies and programs designed to eliminate or reduce the health hazards these negative practices cause. The newsletter keeps the community informed about behavioral factors like nutrition, and the need to eliminate health hazards that evolve from birth through early childhood to adulthood and extending to the next generation [14].

The newsletter is intended to, not only ensure that health information is made more readily available to the community, but also ensure that results from the study and other research studies are presented using community-friendly terminology, with the overall goal of effective utilization by the community. Articles published in the newsletter are designed to provide information to JHS participants and the community on how to take the best care of themselves and their conditions, how to improve their overall health, and how to reduce their chances of developing complications. In addition, the newsletter publishes case histories, public health reports, exclusive surveys, news sections, consultant columns and new health-improving strategies and products [18]. The partnership established between CORC and the JHS community has served to build bridges and mend fences between academia, families, and the community. The efforts by the CORC to sustain the interactions and collaborations with the community through the CHAN has empowered the members of the JHS community to develop new skills, adopt new attitudes, and acquire knowledge that would enable them to become more effective and more successful. True partnerships are developed based on trust, and survival is based on trust. The partnership between the JHS CORC and the CHAN was built on the desire to erect and sustain an environment that can stimulate conditions conducive to safety, learning, healthy behaviors, and good health. The partners continue to strive to ensure the survival of the rich and supportive environment that is building the enthusiasm among community members to contribute.

## 4. Discussion

The impetus for forming the academic-community collaborative partnership emanated from a small group of community members seeking answers for a particular problem-the well documented cardiovascular disease and obesity prevalence among African Americans in Mississippi. CORC and the CHAN aspired to build a culture of health by working together. The accomplishments described by the Jackson Heart Study (JHS) Community Outreach Center (CORC) is a testament that community commitment is more than an expression, a thought, or an idea. In true CBPR fashion, CORC began this collaboration with the expectation that the academic-community partnership would result in real action that involves active planning and participation by our community partners. The Strategic Planning Retreat and subsequent collaborations between CORC and its community partners illustrated that the partners became engaged in real activities where CORC provided resources and services to the community where needed. CORC’s goal is to continue to inspire, motivate, and facilitate community involvement in research, health education, and health promotion to fulfill its goal to empower the local communities through a wide array of education and information initiatives.

CORC’s goal is to improve health status by working closely with the CHAN in the three counties of Hinds, Madison, and Rankin to foster research, mentoring, teaching, service, outreach, capacity-building, and leadership. In order to reduce health disparities, there must be a long-term commitment to transform the traditional academic/researcher/community relationship into a more collaborative partnership where community partners can be respected and their input and contributions solicited, valued, adopted, and utilized. It is evident that health disparities affect the daily lives and experiences of individuals and families, and that negative impact can certainly affect entire communities in the long run. Elevating the CHAN contributions regarding elimination of risk factors and health disparities from being simply a consumer of information to one of equal partner leading the way and contributing to developing solutions can help to expedite the discovery of meaningful alternatives that ordinarily would be kept in obscurity.

## 5. Conclusions

The purpose of this study was to highlight the impact of the multilevel strategies employed by the JHS CORC to build trust and capacity within an academic-community partnership, a primary feature of the JHS, the largest investigation of causes of CVD in an African-American population. The key components of the JHS CORC’s strategies underscored in this study exemplify multilevel strategies that are interrelated and linked to the major CBPR concepts. One such component is the theoretical model AJA Six-Facet Model of Community Engagement that builds the foundation for developing continuing engagement, outreach, and collaboration with the community partners. Another key component is the implementation of a logic model to guide the overall scientific integrity of the academic-community partnership ensuring that knowledge acquired could lead to action that could inspire lifestyle, behavioral, and structural change on an individual basis, as well as community-wide. 

The AJA model and the logic model developed can stimulate community-engaged research and community outreach for the purpose of health education and health promotion because they are designed to facilitate long term sustainability and increased capacity of the CHANs so that they can share knowledge with other CHANs and other community members empowering them to do the work themselves. Future studies that aspire to reduce health disparities and adverse health in the at-risk populations, such as African American or other minority participants, can benefit from the strategies observed in the community engagement and outreach efforts of the JHS since these strategies and approaches have contributed to the successes and durability of the JHS research endeavors. 

The strategies utilized by the JHS CORC can be used as a basis for rethinking community outreach and engagement, particularly in hard to reach populations, such as minority and rural populations. The JHS CORC utilizes its resources and intellectual capital to build research capacity to protect the community’s health and environment, while at the same time promoting policy building/policy changing capacity. The JHS CORC is increasing capacity among the CHANs and their communities to facilitate a community wide assault on risk factors for development of cardiovascular and other chronic diseases as well as negative behavior practices. The rich collaborations that have ensued through this fully engaged academic-community partnerships can facilitate action and policy change where needed, as Mississippi struggles to combat the unequal burden of the prevalence of obesity and cardiovascular diseases. The strategies described in this account serve as an example of how multilevel strategies targeting, high risk, vulnerable populations and communities can be implemented to begin the process of reversing the epidemic of CVD in African Americans.
